# Supplementary material for: Public participation in healthcare safety: A tripartite evolutionary game model with evidence from diverse international cases
Source: PLoS One. 2026 Jan 16;21(1):e0339304. doi: 10.1371/journal.pone.0339304 (PMC12810857; doi:10.1371/journal.pone.0339304)
Supplement: Attachment — Submitted filename: Review Comments-PLOS1.docx [file pone.0339304.s001.docx]

**Title:** *Public Participation in Smart Healthcare Safety Risk Supervision: Insights from a Tripartite Evolutionary Game and Tuberculosis Case Analysis*

Let me commend the authors for their work. The manuscript presents a theoretically robust and policy-relevant contribution to healthcare risk supervision. Its innovative use of evolutionary game theory and empirical validation are commendable. Below are the strengths of the manuscript.

**1. Strengths**

1. **Novelty and Theoretical Contribution**:
   - The study introduces a tripartite evolutionary game model to analyze public participation in healthcare risk supervision, filling a gap in existing literature. The integration of game theory with smart healthcare systems is innovative.
   - The use of tuberculosis (TB) as a case study provides concrete relevance to public health policy, particularly in low-compliance settings.
2. **Methodological Rigor**:
   - The model construction is mathematically sound, with clear assumptions, replication dynamics, and stability analysis using Jacobi matrices and Lyapunov’s first method.
   - Numerical simulations (e.g., XGBoost, SHAP) and real-world data validation strengthen the empirical robustness of the findings.
3. **Practical Implications**:
   - The study offers actionable policy recommendations, such as enhancing exposure mechanisms and adjusting penalty thresholds, which are valuable for healthcare regulators.
   - The emphasis on public participation aligns with global trends in democratizing healthcare governance.
4. **Comprehensive Analysis**:
   - Sensitivity analyses on key parameters (e.g., medical risk, penalty intensity, exposure rate) provide nuanced insights into system dynamics.
   - Visualizations (e.g., 3D plots, phase diagrams) effectively illustrate evolutionary trends and equilibrium states.

#### **These strengths, notwithstanding, some weaknesses must be addressed as follows:**

1. **Data and Generalizability**:
   - The reliance on aggregated secondary data (AlSahafi et al., 2019) limits the granularity of analysis. The model’s validation would benefit from primary data or multi-country datasets to assess cross-cultural applicability.
   - The TB case study, while relevant, may not fully represent other healthcare risks (e.g., chronic diseases, pandemics).
2. **Model Assumptions**:
   - **Bounded Rationality**: The assumption of "limited rationality" for all agents (public, institutions, regulators) is plausible but oversimplifies complex decision-making (e.g., cultural, socioeconomic factors).
   - **Static Parameters**: Key variables (e.g., penalty intensity, exposure rate) are treated as fixed, whereas real-world policies often adapt dynamically.
3. **Ethical and Implementation Challenges**:
   - The proposal to increase penalties and public exposure mechanisms raises ethical concerns (e.g., privacy violations, stigmatization of non-compliant institutions). These are not adequately addressed.
   - The model does not account for potential unintended consequences, such as regulatory capture or public distrust due to over-penalization.
4. **Technical Clarity**:
   - The manuscript is dense with mathematical notation, which may hinder readability for non-technical audiences. A summary table of variables or a glossary would improve accessibility.
   - Some figures (e.g., Fig 3, Fig 8) lack clear captions or annotations, making interpretation difficult.

#### **3. Recommendations for Improvement**

1. **Expand Data Sources**:
   - Incorporate primary data or diverse case studies (e.g., COVID-19, antibiotic misuse) to validate the model’s broader applicability.
2. **Dynamic Modeling**:
   - Introduce time-varying parameters or stochastic elements to reflect real-world policy adaptability and uncertainty.
3. **Ethical and Policy Nuance**:
   - Discuss trade-offs of proposed interventions (e.g., balancing transparency with privacy) and suggest safeguards.
   - Address potential resistance from medical institutions to punitive measures.
4. **Clarity and Presentation**:
   - Simplify mathematical sections with intuitive explanations or appendices.
   - Improve figure labels and provide standalone interpretations (e.g., "Fig 6 shows that medical risk is the most influential parameter").
5. **Future Research Directions**:
   - Explore multi-level governance (e.g., local vs. national regulators) or hybrid supervision models (e.g., AI-assisted public reporting).
