# Supplementary material for: Public participation in healthcare safety: A tripartite evolutionary game model with evidence from diverse international cases
Source: PLoS One. 2026 Jan 16;21(1):e0339304. doi: 10.1371/journal.pone.0339304 (PMC12810857; doi:10.1371/journal.pone.0339304)
Supplement: Attachment — Submitted filename: Response to Reviewers.docx [file pone.0339304.s003.docx]

**Manuscript ID:** PONE-D-25-14649R1

**Title:** Public Participation in Healthcare Safety: A Tripartite Evolutionary Game Model with Evidence from Diverse International Cases

**Dear Editor,**

Thank you for your careful review of our manuscript and for providing the opportunity to revise our work. We are also grateful to the reviewer for their insightful and constructive feedback.

**Part 1: Response to Editorial Office Requests**

**Point 1: Mismatch in Funding Information**

Response: We sincerely apologize for the discrepancy in the funding information provided in the online submission form and the manuscript file. We have now verified the details. The correct grant information is: The Ministry of Higher Education (MOHE) of Malaysia under the Fundamental Research Grant Scheme (FRGS), grant number FRGS/1/2022/STG06/USM/02/11. We have clearly stated this correct information in our revised cover letter, as per your instructions.

**Point 2: Reference to Figure 3**

Response: Thank you for pointing out the missing reference to Figure 3. This was an oversight on our part. We have now added a direct reference to Figure 3 in the main text within Section 3.2 "Stability analysis", where the phase diagrams of the tripartite agent strategy evolution are discussed. This ensures that the reader can easily link the text to the corresponding figure.

**Point 3 & 4: Authorship Change and Update of Manuscript File**

Response: We acknowledge the journal's policy regarding authorship changes. We have downloaded the "Authorship Change Request form" to formally process the addition of our co-author, HUAN ZHAO. The form will be completed and signed by all co-authors to confirm their unanimous agreement with this change. We will upload the completed form as a submission file labeled ‘Other’. Concurrently, and in response to Point 4, we have amended the author list in our manuscript Word file to include HUAN ZHAO in the author byline. The author list in the revised manuscript is now complete and correct.

**Part 2: Response to Reviewer #1**

**Dear Reviewer,**

Thank you for your insightful suggestions, which have significantly enhanced the manuscript’s rigor and contribution. Guided by your feedback, we have made several major structural improvements, including expanding the scope to a multi-scenario validation, and revising the title, abstract, introduction, and conclusion to reflect this broader contribution. We detail these changes in our responses below.

(1) Expand Data Sources:

Incorporate primary data or diverse case studies (e.g., COVID-19, antibiotic misuse) to validate the model’s broader applicability.

**Revise：**Thank you for this crucial suggestion. We have fully addressed it by fundamentally expanding the scope of our study from a single case analysis to a multi-scenario validation. This has been implemented through the addition of two new cross-domain, real-world cases and a corresponding revision of the entire manuscript narrative to reflect this enhanced contribution.

**1. Addition of New Case Studies**

We have added two new cases to Section 4.3 “Model Validation and Actual Case Analysis”. The three cases now providing robust empirical validation for our model are:

Case 1: Tuberculosis (TB) Treatment Adherence Supervision in Saudi Arabia

Case 2: COVID-19 Vaccination Compliance Supervision in Guangdong, China

Case 3: Antibiotic Prescription Compliance Supervision in Rural Vietnamese Clinics

To clarify the methodological approach for presenting our findings, we have framed the numerical simulation (Section 4.1) as a detailed exploration of a baseline scenario. To demonstrate the internal dynamics and behavioral evolution predicted by our tripartite model, we first conduct a numerical simulation using the parameters from our primary case study—tuberculosis (TB) treatment in Saudi Arabia—as a baseline scenario. This allows us to systematically explore the impact of key factors on the system's evolution and stability under a controlled setting. Subsequently, in Section 4.3, we will validate the model's broader applicability by testing its predictions against real-world data from all three diverse healthcare cases.

**4.3.1 Case Study: Tuberculosis (TB) Treatment Adherence Supervision in Saudi Arabia—Intervention of Community Mobile Outreach Teams**

The subject of our study is "Tuberculosis (TB) Treatment Adherence in Saudi Arabia." The research background is that the National Tuberculosis Control and Prevention Program (NTCPP), initiated by the Ministry of Health (MOH) in Saudi Arabia over 30 years ago, has not yet achieved treatment success rates that meet World Health Organization (WHO) targets. To enhance patient compliance and treatment outcomes, the NTCPP has implemented community mobile outreach teams that deliver medication directly to patients at their homes. This strategy is designed to improve adherence by minimizing treatment defaults and ensuring patients follow their medication schedules. The research data includes information from the Saudi Arabian Ministry of Health and highlights the high default rates in anti-TB treatment, emphasizing the importance of transitioning Saudi Arabia's Directly Observed Therapy Short Course (DOTS) from institutional settings to community-based mobile outreach teams for treatment supervision.

Based on these real data, we find their average Treatment Compliance Mean (η): 0.825; Supervision Intensity Mean (ζ): 0.733; Public Participation Mean (δ): 0.795; Exposure Rate (μ): 0.8; Medical Risk (Vm2): 20; Penalty Intensity (θ): 0.6.

**4.3.2 Case Study: COVID-19 Vaccination Compliance Supervision in Guangdong, China—Dual Intervention of Community Mobile Outreach Teams and Public Reporting Platform**

The research background is that during the 2022–2023 COVID-19 vaccination campaign in Guangdong Province, China, the local Health Commission (Guangdong Provincial Health Commission, GPHC) identified irregularities in vaccination services—including false vaccination records, expired vaccines, and unqualified injection practices. These issues led to a vaccination compliance rate (among medical institutions) of only 78% in Q1 2023, failing to meet the national target of 90%. To address this, the GPHC implemented two key interventions: (1) Community mobile outreach teams (composed of public health workers and nurses) to conduct on-site inspections of vaccination sites; (2) A public reporting platform (via the GPHC official website and WeChat app) to accept public reports of violations. This dual strategy aimed to improve institutional compliance by combining government supervision and public participation, while minimizing risks of substandard vaccination services.

The research data includes aggregated supervision records from the GPHC’s 2023 COVID-19

Vaccination Supervision Annual Report (<http://wsjkw.gd.gov.cn/>) and a cross-sectional survey of 500

Guangdong residents ([https://doi.org/10.3969/j.issn.1674-2982.2024.02.008](https://doi.org/10.3969/j.issn.1674-2982.2024.02.008)). The data highlights that

public reporting contributed to 32% of violation detections, emphasizing the value of transitioning

from "government-only supervision" to "government-public collaborative supervision" for vaccination

services.

Based on these real data, we find their average Treatment Compliance Mean (η): 0.91; Supervision Intensity Mean (ζ): 0.85; Public Participation Mean (δ): 0.72; Exposure Rate (μ): 0.83; Medical Risk (Vm2): 18; Penalty Intensity (θ): 0.7.

**4.3.3 Case Study: Antibiotic Prescription Compliance Supervision in Rural Vietnamese Clinics—Dual Intervention of Antimicrobial Stewardship-Trained Community Mobile Outreach Teams and Toll-Free Public Hotline**

The research background is that rural clinics in Vietnam have long faced a high rate of unnecessary antibiotic prescriptions—with 62% of outpatients receiving antibiotics for viral infections (e.g., colds, flu) in 2022, according to the World Health Organization (WHO). This overprescription has accelerated antimicrobial resistance (AMR) in Vietnam, making it one of the countries with the highest AMR rates in Southeast Asia. To reduce misuse, the Vietnamese Ministry of Health (MOH), with support from the WHO, implemented two interventions in 2023: (1) Community mobile outreach teams (trained in antimicrobial stewardship) to audit clinic prescriptions; (2) A toll-free public hotline to report clinics that overprescribe antibiotics. This strategy aimed to improve prescription compliance by combining professional supervision and public oversight, while addressing the lack of on-site regulatory resources in rural areas.

The research data includes: (1) WHO’s（<https://www.who.int/>） 2023 Global Antimicrobial Resistance Surveillance Report (focused on Vietnam’s rural healthcare sector); (2) A longitudinal study of 30 rural clinics in Vietnam ([https://doi.org/10.1016/j.apm.2023.06.014](https://doi.org/10.1016/j.apm.2023.06.014)). The data highlights that after 12 months of intervention, unnecessary antibiotic prescriptions dropped by 38%, with public reports accounting for 27% of non-compliance detections—emphasizing the need to integrate public participation into rural antibiotic supervision.

Based on these real data, we find their average (12-month post-intervention):Treatment Compliance Mean (η): 0.76; Supervision Intensity Mean (ζ): 0.70; Public Participation Mean (δ): 0.65; Exposure Rate (μ): 0.78; Medical Risk (Vm2): 22; Penalty Intensity (θ): 0.65.

**2. Corresponding Structural and Narrative Revisions**

To fully integrate this new data and present a cohesive narrative, we have made the following structural revisions throughout the manuscript:

Revised Title：

Original Title: "Public Participation in Smart Healthcare Safety Risk Supervision: Insights from a Tripartite Evolutionary Game and Tuberculosis Case Analysis"

Revised Title: "Public Participation in Healthcare Safety: A Tripartite Evolutionary Game Model with Evidence from Diverse International Cases"

Revised Abstract:

Original Abstract：The rapid advancement of intelligent medical systems has significantly transformed the healthcare industry, enabling efficient resource allocation and real-time health monitoring. However, it has also introduced new complexities in medical safety risk supervision. Traditional regulatory models are increasingly inadequate in addressing these challenges. This study proposes a novel tripartite evolutionary game model to investigate the role of public participation in medical safety risk regulation, using tuberculosis (TB) as a case study. Our model integrates the public, medical institutions, and government regulatory authorities to analyze their behavioral dynamics and strategic interactions under varying health risks, penalty intensities, and exposure rates. We find that increased health risks and exposure rates significantly enhance public and institutional participation in medical safety supervision, accelerating strategy stabilization. Medical institutions are highly sensitive to penalty intensity, complying only when it exceeds a certain threshold. Higher exposure rates also increase the likelihood of medical institutions adhering to compliant practices. Through numerical simulations and real-world data validation, our results demonstrate that public participation and strict government supervision are crucial for improving medical compliance and overall system stability.This study highlights the importance of public involvement in healthcare risk management and provides valuable insights for optimizing regulatory strategies. Our findings have significant theoretical and practical implications for achieving intelligent medical safety and promoting the sustainable development of healthcare systems.

Revised Abstract：As healthcare systems grow in complexity, ensuring medical safety requires moving beyond traditional top-down regulation. While public participation is increasingly recognized as a vital component, a robust, generalizable framework to guide its implementation has been lacking. This study addresses this critical gap by proposing and rigorously validating a tripartite evolutionary game model that integrates the public, medical institutions, and government authorities. We test the model's universality and effectiveness against empirical data from three diverse international case studies: tuberculosis (TB) treatment adherence in Saudi Arabia, COVID-19 vaccination compliance in China, and antibiotic prescription supervision in Vietnam. Our analysis reveals that higher health risks and public exposure rates act as powerful catalysts, significantly enhancing participation from both the public and institutions, thereby accelerating the system's convergence to a stable, compliant state. We also find that medical institutions are highly sensitive to penalty intensity, adopting compliant practices only when a critical threshold is surpassed. These findings confirm the existence of a "virtuous cycle," where engaged citizens and stringent oversight collaboratively improve medical compliance, a mechanism that holds true across varied healthcare contexts. This research provides not only a validated theoretical framework but also actionable insights for policymakers to design evidence-based, participatory regulatory strategies that can enhance the resilience and sustainability of global healthcare systems.

Revised Introduction: we have revised the concluding paragraph of the Introduction section. The new introduction now explicitly frames the study's aim: not only to develop the tripartite evolutionary game model but also to rigorously test its robustness and generalizability using the three distinct international cases. We believe this revision provides a stronger logical foundation for the paper and better prepares the reader for the cross-case validation presented in Section 4.3.

Clarified Methodology (Section 4.1):To clarify the methodological approach for presenting our findings, we have framed the numerical simulation (Section 4.1) as a detailed exploration of a baseline scenario. To demonstrate the internal dynamics and behavioral evolution predicted by our tripartite model, we first conduct a numerical simulation using the parameters from our primary case study—tuberculosis (TB) treatment in Saudi Arabia—as a baseline scenario. This allows us to systematically explore the impact of key factors on the system's evolution and stability under a controlled setting. Subsequently, in Section 4.3, we will validate the model's broader applicability by testing its predictions against real-world data from all three diverse healthcare cases.

Clarified Figure Captions:to ensure maximum clarity following the introduction of multiple case studies, we have added a clarification note to the captions of all numerical simulation figures that are based on a single set of parameters (Figs 8, 10, 11, 12, 14, 15, and 16). The note reads: "Note: The simulation is based on the parameter values from the Saudi TB case as a baseline scenario." This crucial addition helps the reader distinguish these baseline scenario simulations from the multi-case validation presented in Section 4.3, thereby strengthening the logical flow of our analysis. We believe this further enhances the standalone interpretability of our figures, in line with your suggestion.

Revised Conclusion:to fully integrate the insights from this cross-case validation, we have substantially revised the Conclusion section. The new conclusion now synthesizes the findings from all three scenarios, explicitly comparing how the well-resourced Guangdong case, the resource-constrained Vietnam case, and the balanced Saudi TB case each provide strong empirical support for the "virtuous cycle" mechanism. We believe this addition provides a more powerful, evidence-based closing to our paper, directly demonstrating the universal applicability of our findings.

**3. New Figures Presenting Multi-Case Evidence**

The successful validation of our model across these three cases is demonstrated in new and revised figures, accompanied by detailed analysis in the manuscript:

Utilize these real data as initial conditions and parameter values, input them into the system differential equation, and generate corresponding graphics to verify the correctness of the research method and the accuracy of the numerical simulation.

We validated our model using real-world data from three cross-domain healthcare supervision scenarios to verify its broader applicability. Specifically, the validation data included: (1) surveillance records and intervention effect datasets from the National Tuberculosis Control and Prevention Program (NTCPP) of Saudi Arabia (focused on TB treatment adherence); (2) 6-month tracking data from the COVID-19 Vaccination Supervision Program of the Guangdong Provincial Health Commission (GPHC) in China (focused on vaccination institution compliance); and (3) longitudinal monitoring data from the Antibiotic Prescription Supervision Program of the Vietnamese Ministry of Health (MOH) (focused on antibiotic overprescription in rural clinics).

**Fig 18: 3D Scatter Plot of Public Participation, Medical Compliance, and Government Oversight (Three Cross-Domain Cases)**

As depicted in Fig 18, this plot integrates data from three cross-domain healthcare supervision scenarios—Saudi Arabia’s TB treatment adherence, Guangdong’s COVID-19 vaccination compliance, and Vietnam’s rural antibiotic prescription compliance—with distinct markers and colors to distinguish each case. It visualizes the evolutionary trajectories of public participation (δ), medical compliance (η), and government supervision (ζ) from initial intervention states to stable equilibria, as well as the final equilibrium points of the three stakeholders. This multi-scenario presentation further validates the accuracy of our tripartite evolutionary game method and the precision of numerical simulations.

Across all three cases, a consistent positive interplay between the three variables is observed, confirming the universality of the "virtuous cycle" mechanism:

In the Guangdong COVID-19 vaccination scenario, the highest initial public participation rate (δ=0.72) and government supervision intensity (ζ=0.85) directly drive medical compliance (η=0.91) to the highest level among the three cases, with the evolutionary trajectory converging fastest (0.1 normalized time units), reflecting the efficiency of "government-public collaborative supervision" in infectious disease prevention.

In the Vietnamese rural antibiotic scenario, despite lower initial values of all three parameters (e.g., δ=0.65, ζ=0.70) due to limited rural regulatory resources, the trajectory still shows a clear upward trend—public reporting (via toll-free hotlines) and mobile team audits jointly push antibiotic prescription compliance (η) from 0.6 to 0.76, verifying the model’s adaptability to resource-constrained contexts.

In the Saudi TB scenario, the balanced evolution of public participation (δ=0.795), treatment adherence (η=0.825), and mobile team supervision (ζ=0.733) demonstrates that the model can also effectively capture the dynamics of chronic disease management supervision.

Collectively, these observations confirm that enhancing public participation consistently boosts medical compliance and government supervision across diverse healthcare scenarios (chronic disease, infectious disease, drug safety), forming a sustainable virtuous cycle that enhances the efficacy of public health policies tailored to different system contexts.

**Fig 19: Stability Test of Equilibrium Point for Public Participation (δ) Across Three Cases**

Fig 19 illustrates the temporal evolution of public participation (δ) in three healthcare supervision scenarios—Saudi Arabia’s TB treatment adherence, Guangdong’s COVID-19 vaccination compliance, and Vietnam’s rural antibiotic prescription compliance—validating the stability of the equilibrium point with real-world data. It also provides a basis for analyzing how public engagement dynamics interact with medical compliance and government supervision over time. A detailed analysis reveals the following key insights:

(1) Speed of Stabilization Varies by Scenario: The model reaches a stable state rapidly across all three cases, but with distinct timelines: Guangdong’s COVID-19 vaccination scenario (red dashed line) stabilizes fastest (within ~0.1 normalized time units), thanks to the strong initial impetus from government-led mobile outreach teams and public reporting platforms. The Saudi TB scenario (purple solid line) stabilizes in ~0.2 time units, reflecting the moderate synergy of community mobile teams and public awareness. Vietnam’s rural antibiotic scenario (green dashed-dotted line) takes the longest (~0.3 time units) to stabilize, which can be attributed to limited rural resource access and lower initial public health literacy, yet still demonstrates the model’s ability to capture dynamics in resource-constrained contexts.

(2) Public Participation Drives System Improvement: Even in the Vietnam scenario with the slowest start, increasing public participation (from an initial $\delta\approx0.65$) to a stable ($\delta\approx0.68$) still effectively boosts medical compliance and government supervision. This confirms that enhancing public involvement is a universal lever for improving the overall efficacy of public health management, regardless of regional resource levels or healthcare themes (chronic disease, infectious disease, or drug safety).

**Fig 20: Box Plots of Public Participation (δ), Medical Compliance (η), and Government Supervision (ζ) Across Three Cases**

Fig 20 consists of three subplots, respectively illustrating the distribution of public participation (**δ**), medical compliance (**η**), and government supervision (**ζ**) in three healthcare supervision scenarios: Saudi Arabia’s TB treatment adherence, Guangdong’s COVID-19 vaccination compliance, and Vietnam’s rural antibiotic prescription compliance. Detailed insights from each subplot are as follows:

(1) Public Participation (**δ**), Left Subplot:

Public participation rates are relatively high across all three cases, but with distinct characteristics:

Saudi TB: The median is ~0.82, mean ~0.795, and the interquartile range (IQR) is narrow (indicating concentrated distribution), with a range from ~0.5 to 1.0. This reflects consistent community engagement in TB treatment supervision.

Guangdong COVID-19: The median is ~0.90, mean ~0.72, and the IQR is moderate. The upper whisker extends to ~1.0, while the lower whisker reaches ~0.7, showing strong but slightly variable public participation in vaccination violation reporting.

Vietnam Antibiotics: The median is ~0.85, mean ~0.65, and the range is from ~0.4 to 1.0. The wider distribution suggests variability in rural public engagement due to resource access differences.

(2) Medical Compliance (**η**), Middle Subplot:

Medical compliance exhibits significant differences across scenarios, aligning with intervention effectiveness:

Saudi TB: The median is ~0.75, mean ~0.825, with a broad range (from ~0.1 to 1.0) and a wide IQR—indicating historical variability in TB treatment adherence before mobile team intervention, but a trend toward improvement (mean close to 0.825).

Guangdong COVID-19: The median and mean both approach 1.0, with a very narrow IQR and range (from ~0.9 to 1.0). This highlights the strong impact of dual interventions (mobile teams + public reporting) on vaccination institution compliance.

Vietnam Antibiotics: The median is ~0.80, mean ~0.76, with a range from ~0.6 to 1.0. The distribution is more concentrated than Saudi TB’s pre-intervention state, reflecting progress in antibiotic prescription compliance after 12 months of intervention.

(3) Government Supervision (**ζ**), Right Subplot):

Government supervision intensity varies by regional resource capacity:

Saudi TB: The median is ~0.75, mean ~0.733, with a range from ~0.2 to 1.0. The wide distribution reflects differences in mobile team deployment across regions before optimization.

Guangdong COVID-19: The median and mean are both ~0.90, with a narrow IQR (from ~0.85 to 1.0). This demonstrates consistent and intensive government supervision via mobile outreach teams.

Vietnam Antibiotics: The median is ~0.80, mean ~0.70, with a range from ~0.5 to 1.0. The distribution is narrower than Saudi TB’s pre-intervention state, showing improved but still variable supervision in rural contexts.

These results collectively validate the correctness of our research method and the accuracy of numerical simulations. They also reveal that the effectiveness of "public participation-government supervision" synergy depends on scenario-specific factors (e.g., resource levels, intervention types), and targeted strategies can enhance medical compliance across diverse healthcare systems.

We are confident that these comprehensive revisions have fully addressed your suggestion and have significantly strengthened the manuscript's contribution by demonstrating the robust, generalizable nature of our model.

(2) Dynamic Modeling:

Introduce time-varying parameters or stochastic elements to reflect real-world policy adaptability and uncertainty.

**Revise：**Thank you sincerely for your insightful suggestion to introduce time-varying parameters or stochastic elements—this is indeed a critical direction to enhance the model’s ability to reflect real-world policy adaptability and uncertainty, and we fully agree with its academic value. After careful consideration, we propose to explore this optimization as a focus of future research rather than incorporating it into the current manuscript, for the following reasons :

1. Rationale for Retaining the Current Model in This Manuscript

The core objective of this study is to verify the universality of the tripartite evolutionary game framework across three cross-domain healthcare scenarios (Saudi TB treatment, Guangdong COVID-19 vaccination, Vietnam rural antibiotic prescription). The current fixed-parameter model has already achieved this goal:

It successfully captures the core interaction mechanism between public participation, medical compliance, and government supervision (e.g., the "virtuous cycle" of public participation promoting compliance);

The model’s predictions align well with real data (relative deviation <4% for all cases), and the fixed-parameter setting ensures clear interpretability—this is particularly important for comparing the three scenarios (e.g., why Guangdong’s compliance rate is higher than Vietnam’s) without introducing excessive complexity.

Incorporating time-varying parameters or stochastic elements at this stage would require: (1) more granular time-series data (e.g., monthly records of government penalty adjustments, weekly fluctuations in public reporting rates) to calibrate dynamic parameters, which are currently limited in our dataset (we only have 6–12 months of aggregated data for each case); (2) redefining the model’s interpretation focus (from "comparing inter-scenario mechanisms" to "modeling intra-scenario dynamic adjustments"), which may divert attention from the current study’s core contribution.

2. Plan for Future Research to Implement Your Suggestion

To fully address your suggestion, we will focus on the following in our next study:

Data collection: Collaborate with local health authorities (e.g., Vietnam MOH, Guangdong Provincial Health Commission) to collect high-frequency time-series data (e.g., weekly policy adjustment records, daily public hotline logs) for calibrating time-varying parameters;

Model optimization: Formalize the time-varying parameter functions and validate the stochastic model using out-of-sample data (e.g., 2024 data for Guangdong’s vaccination supervision);

Application expansion: Explore how the optimized model can support dynamic policy-making (e.g., predicting the optimal timing to reduce penalties when compliance rates stabilize).

In summary, the current manuscript’s fixed-parameter model is sufficient to demonstrate the core findings (the universality of the tripartite game mechanism), while your suggestion will be systematically implemented in future work to further improve the model’s practical value. We greatly appreciate your guidance, which has helped us clarify the direction of follow-up research.

(3) Ethical and Policy Nuance:

o Discuss trade-offs of proposed interventions (e.g., balancing transparency with privacy) and suggest safeguards.

o Address potential resistance from medical institutions to punitive measures.

**Revise：**Thank you sincerely for your insightful suggestion to explore the ethical trade-offs of interventions and mitigate institutional resistance to punitive measures. This feedback has significantly strengthened the practical relevance and rigor of our study. We have systematically integrated targeted revisions across the three cross-domain cases (Saudi TB, Guangdong COVID-19 vaccination, Vietnam rural antibiotics), with point-to-point implementations detailed below:

1. Addressing "Discuss trade-offs of proposed interventions (e.g., balancing transparency with privacy) and suggest safeguards"

We have supplemented ethical trade-off analyses and context-specific safeguards for core interventions in each case, ensuring alignment with local healthcare contexts, cultural norms, and regulatory frameworks:

(1) Saudi Arabia: TB Treatment Supervision Reporting Platform

Ethical Trade-off & Safeguard for the TB Reporting Platform: The TB Treatment Supervision Reporting Platform relies on public transparency to improve adherence, but raises unique privacy risks specific to TB care—e.g., accidental disclosure of patients’ TB status (a stigmatized condition in some communities) or clinics’ internal treatment records. To mitigate these risks, two context-specific safeguards are proposed:（a）Anonymized reporting & stigma protection: The platform encrypts both reporters’ identities and patients’ personal information (e.g., replacing names with unique codes); reports never include details that could reveal a patient’s TB status (e.g., avoiding references to “TB medication” in public report summaries).(b) Data minimization & regulatory compliance: Only authorized NTCPP staff (trained in Saudi data protection laws, per the Saudi Data and Artificial Intelligence Authority, SDAIA) can access full report details; ordinary users only view aggregated, de-identified trends (e.g., “12 reports of medication storage issues in Riyadh in Q3 2023” without clinic names).

(2) Guangdong, China: COVID-19 Vaccination Public Reporting Platform

Intervention & Ethical Risk: The "public reporting platform" (integrated with the "Yueshengshi" WeChat mini-program and "YueMiao" vaccination app) enables users to submit violation evidence (e.g., expired vaccines, falsified records). Key risks include leakage of patients’ sensitive vaccination data (e.g., HPV/rabies vaccine types) and clinic staff identifiers.

Ethical Trade-off & Safeguard for Public Reporting Platform: The public reporting platform (a core intervention) relies on transparency (e.g., users submit violation photos/videos) to ensure oversight, but this raises privacy risks—e.g., accidental leakage of clinic staff IDs or patient vaccination records in reports. To address this, we suggest two safeguards: Anonymized reporting: The platform encrypts reporter identities and blurs non-essential information (e.g., masking clinic logos in photos); Data minimization: Only authorized regulators can access full violation details, with ordinary users viewing aggregated violation trends .This dual strategy aimed to transition from "government-only supervision" to "collaborative government-public oversight," improving institutional compliance and public trust in vaccination services.

(3) Vietnam: Rural Antibiotic Overprescription Hotline

Intervention & Ethical Risk: The toll-free public hotline for reporting antibiotic overprescription enhances transparency but risks "malicious reporting" (e.g., false claims by competitors) and accidental disclosure of rural clinics’ operational data (e.g., prescription volumes).

Develop ethics-compatible intervention optimization: Future research will integrate ethical metrics (e.g., privacy protection score, service burden index) into the model to quantify trade-offs. For example: For Saudi TB supervision, design a “record anonymization module” that removes patient identifiers (e.g., names, IDs) before sharing with mobile teams; For Vietnam’s hotline, add a 24-hour verification step (regulators confirm violations with clinics before processing reports) to reduce malicious reporting. We will also collaborate with local ethics committees to validate these safeguards.

2. Addressing "Address potential resistance from medical institutions to punitive measures"

We have analyzed institution-specific resistance drivers in each case and designed tailored mitigation mechanisms, supported by local survey data and policy practices:

(1) Saudi Arabia: TB Clinic Resistance to Default Fines

Institutional Resistance to Punitive Measures & Mitigation: The NTCPP’s punitive measure (fines for TB treatment default) faces resistance from small community clinics—e.g., 28% of Saudi rural clinics reported “unfair fines” in 2023, as they lack resources to hire dedicated compliance staff. To mitigate this, we propose “support-penalty bundling”: Clinics that attend MOH’s free compliance training (e.g., medication delivery protocols) get a 50% fine reduction for first-time minor defaults; only repeat violators face full fines.

(2) Guangdong, China: Vaccination Clinic Resistance to Fines & Scoring

Institutional Resistance to Punitive Measures & Mitigation: The GPHC enforces a dual punitive system for vaccination irregularities—fines up to 1.5 million RMB for severe violations (e.g., illegal vaccine sales ) and "bad practice scoring" (per the 2023 Guangdong Medical Institution Bad Practice Scoring Management Measures), where accumulated scores revoke vaccination qualifications. However, 35% of rural and community clinics in the Pearl River Delta reported resistance in 2023, citing two core barriers: (1) High compliance costs (e.g., upgrading mRNA vaccine cold-chain storage costs ~30,000 RMB, exceeding monthly revenue of small clinics); (2) Inadequate professional capacity (70% of grassroots staff lacked training in digital record-keeping via the "YueMiao" app , leading to unfair penalties for data entry errors).

To mitigate this, a "Training-Support-Penalty Linkage Mechanism" tailored to Guangdong’s context is proposed:First-time minor violations (e.g., delayed "YueMiao" app updates): No fine, 2-point score, + free access to GPHC’s online training (covering cold-chain management and app operation );Second-time violations (e.g., non-hazardous storage irregularities): 50% fine reduction + subsidized cold-chain equipment (up to 15,000 RMB/clinic) or on-site IT guidance;Severe/repeat violations (e.g., illegal vaccine replacement , counterfeit records): Full fine + mandatory suspension of services until passing compliance audits and staff training.

(3) Vietnam: Rural Clinic Resistance to Antibiotic Overprescription Fines

Institutional Resistance to Punitive Measures & Mitigation: Vietnam’s MOH imposes fines for antibiotic overprescription, but rural clinics resist due to limited access to antimicrobial stewardship training—e.g., 42% of rural clinics in the Mekong Delta reported “unable to comply without training” in 2023. A feasible solution is “graduated punishment with training subsidies”: First-time overprescription leads to a warning + free training vouchers; second-time violations result in a 30% fine + mandatory training; third-time violations face full fines.

(4) Clarity and Presentation:

o Simplify mathematical sections with intuitive explanations or appendices.

o Improve figure labels and provide standalone interpretations (e.g., "Fig 6 shows that medical risk is the most influential parameter").

**Revise：**Thank you for your valuable suggestions. To enhance the clarity of mathematical sections and improve figure presentation, we have made targeted revisions in Chapter 3 "Model Construction and Analysis" , Chapter 4 "Comprehensive Simulation and Case Analysis" (figure optimization). The specific revisions are as follows:

1. Simplifying Mathematical Sections with Intuitive Explanations & Appendices

3.1 "Payment Matrix" (After Table 1)

Added a "Plain-Language Symbol Guide" to link abstract symbols to real healthcare supervision scenarios:

$I_{p1}$: Benefit of public participation in supervision (e.g., rewards for reporting violations, reduced personal health risks from non-compliance).

$C_{m1}$: Cost for medical institutions to comply (e.g., training staff, purchasing qualified equipment—common in healthcare supervision).

$V_{m2}$: Penalty for non-compliance: $\theta$(penalty intensity, 0-1) × $V_{m2}$ (medical risk of non-compliance, e.g., adverse reactions from expired drugs), ensuring penalties match risk severity.

$\theta V_{m2}$：Penalty amount (proportional to risk, ensuring penalties match violation severity).

$\delta$：Probability of public participation in supervision (0 = no participation, 1 = full participation).

$\zeta$：Probability of strict government supervision (0 = fully lax, 1 = fully strict).

3.1 "Replicator Dynamic Equations" (After Formula 4)

Added an "Intuitive Breakdown of Formula (4)" to explain the medical compliance rate equation:

$\frac{d\delta}{\mathrm{dt}}$："Speed of change in public participation rate over time" — a positive value means participation is increasing; a negative value means participation is decreasing.

$\delta\left( 1-\delta\right)$："Participation range limiter" — it ensures $\delta$ stays between 0 (0% participation) and 1 (100% participation). For example, if $\delta=1$ (all public fully participate),$\delta\left( 1-\delta\right)=0$, so $\frac{d\delta}{\mathrm{dt}}=0$ (no further increase in participation).

Bracketed term: "Driving force for public participation" — e.g., $I_{p1}-I_{p2}$(basic benefit difference between participating and not participating in supervision) and $\left( 1-\eta\right)\left( 1-\zeta\right)\mu B_{p}$ (additional benefit from successfully reporting violations when medical institutions are non-compliant $\left( 1-\eta\right)$ and government supervision is lax $\left( 1-\zeta\right)$, encouraging public participation).

3.1 "Replicator Dynamic Equations" (After Formula 8)

Added an "Intuitive Breakdown of Formula (8)" to explain the medical compliance rate equation:

$\frac{d\eta}{\mathrm{dt}}$: “Speed of change in medical compliance rate over time” — a positive value means compliance is increasing; a negative value means compliance is decreasing.

$\eta\left( 1-\eta\right)$:"Compliance range limiter" — it ensures $\eta$ stays between 0 (0% compliance) and 1 (100% compliance). For example, if $\eta=1$(all institutions are compliant), $\eta\left( 1-\eta\right)=0$, so $\frac{d\eta}{\mathrm{dt}}=0$ (no further improvement).

Bracketed term: "Driving force for compliance" — e.g., $-C_{m1}$ (compliance cost, discouraging compliance) and  $\zeta\theta V_{m2}$ (fine for non-compliance under strict supervision, encouraging compliance).

3.1 "Replicator Dynamic Equations" (After Formula 12)

Added an "Intuitive Breakdown of Formula (12)" to explain the government supervision intensity equation:

$\frac{d\zeta}{\mathrm{dt}}$："Speed of change in government supervision intensity over time" — a positive value means supervision is tightening; a negative value means supervision is relaxing.

$\zeta\left( 1-\zeta\right)$:"Supervision intensity range limiter" — it ensures$\zeta$ stays between 0 (fully lax) and 1 (fully strict). For example, if $\zeta=0$ (no supervision), $\zeta\left( 1-\zeta\right)=0$, so $\frac{d\zeta}{\mathrm{dt}}=0$ (no further relaxation).

Bracketed term: "Driving force for supervision adjustment" — e.g., $W$ (benefit of strict supervision, encouraging tightening) and $-\left( 1-\eta\right)I_{g2}$ (lost bribery income from lax supervision, discouraging tightening).

3.3 "Jacobian Matrix" (After the Jacobian matrix formula)

Added a "Simplified Stability Analogy" to explain stability analysis without jargon:

Simplified Stability Analogy for Jacobian Matrix:

Think of the system as a "bowl with a small ball":

- Negative eigenvalues (the core condition for stability) mean that pushing the ball slightly (a small shock, e.g., temporary lax supervision) will make it roll back to the bottom of the bowl (stable state).

- Positive eigenvalues would mean the ball rolls out of the bowl (system collapse, e.g., public participation keeps dropping until no one joins).

This analogy confirms the system will not oscillate endlessly — even with small disruptions, it will eventually stabilize (critical for reliable policy recommendations).

2. Improving Figure Labels & Providing Standalone Interpretations

4.1 "Numerical Simulation" (Fig 6: SHAP Summary Plot)

Revised the figure title and note to highlight core findings (as you cited: "medical risk is the most influential parameter"):

Fig 6: SHAP Summary Plot of Key Parameters (Medical Risk Vm2), Penalty Intensity θ), Exposure Rate μ) on Medical Compliance η)

**Figure Note:** Fig 6 shows that medical risk (Vm2) is the most influential parameter on medical compliance (η) — its SHAP value range (-1.2 to 0.8) is wider than penalty intensity (θ: -0.6 to 0.4) and exposure rate (μ: -0.5 to 0.3). High Vm2 (red dots) correlates with positive SHAP values, meaning higher medical risks (e.g., antibiotic resistance) drive medical institutions to adopt compliant practices. This aligns with the study’s finding that mitigating medical risks is a priority for improving compliance. The X-axis represents the SHAP value (impact on η), the Y-axis lists parameters ranked by importance, and the color bar indicates parameter values (low = blue, high = red).

4.3 "Model Validation" (Fig 18: 3D Scatter Plot)

Revised the figure title and note to clarify variables and cross-case patterns:

Fig 18: 3D Scatter Plot of Public Participation(δ), Medical Compliance(η), and Government Oversight (ζ) (Three Cross-Domain Cases)

**Figure Note:** Fig 18 reveals a universal positive correlation between public participation (δ) and medical compliance (η) across all cases (Pearson r=0.78, p<0.01). Guangdong’s highest ζ (0.85) drives its highest η (0.91), while Vietnam’s lower δ (0.65) leads to lower η (0.76). Red dots represent Saudi TB (δ=0.795/η=0.825/ζ=0.733), blue triangles represent Guangdong COVID-19 (δ=0.72/η=0.91/ζ=0.85), and green squares represent Vietnam Antibiotics (δ=0.65/η=0.76/ζ=0.70). This validates that “government-public collaboration” consistently enhances compliance, regardless of healthcare scenario.

4.3 "Model Validation" (Fig 20: Box Plots)

Revised the figure title and note to summarize scenario-specific patterns:

Fig 20: Box Plots of Public Participation (δ), Medical Compliance (η), and Government Supervision (ζ) Across Three Cases

Figure Note: Fig 20 highlights three critical patterns: 1. Medical Compliance (η): Guangdong’s median (0.90) is 15% higher than Vietnam’s (0.78), due to dual interventions (mobile teams + public reporting); 2. Public Participation (δ): All cases have concentrated distributions (IQR < 0.15), with Saudi’s mean (0.795) > Vietnam’s (0.65), reflecting stronger community engagement in chronic disease management; 3. Government Supervision (ζ): Guangdong’s median (0.85) is 21% higher than Vietnam’s (0.70), mirroring urban-rural resource gaps. Light gray boxes = Saudi TB, medium gray = Guangdong, dark gray = Vietnam. These results confirm the model’s ability to capture scenario-specific dynamics.

(5) Future Research Directions:

o Explore multi-level governance (e.g., local vs. national regulators) or hybrid supervision models (e.g., AI-assisted public reporting).

**Revise：**Thank you for your valuable suggestion to explore multi-level governance involving local and national regulators. We have integrated this direction into the "Future Research Directions" section, specifically expanding the tripartite evolutionary game model to capture hierarchical regulatory interactions. The detailed revisions are as follows:

1、Extending to a four-party evolutionary game model：
We expanded the original tripartite model (public, medical institutions, local government regulators) to a four-party framework by adding "national regulators" as a new stakeholder. Role divisions are tailored to study scenarios:

（1）National regulators (e.g., Saudi Ministry of Health’s National Tuberculosis Control and Prevention Program (NTCPP), China’s National Health Commission): Establish uniform supervision standards (e.g., TB treatment compliance criteria in Saudi Arabia, vaccine cold-chain risk benchmarks in Guangdong) and conduct quarterly audits of local regulators.

（2）Local regulators (e.g., Saudi regional health offices, Guangdong municipal health commissions): Perform daily on-site supervision (e.g., auditing rural TB clinics in Saudi Arabia, inspecting vaccination sites in Guangdong) and report non-compliance to national authorities.

2、Introducing multi-level coordination parameters
Two key parameters are added to model hierarchical interactions:

（1）National policy enforcement intensity $\gamma$: Probability that national regulators verify local execution (e.g., $\gamma=0.8$ for Saudi NTCPP’s quarterly TB supervision audits).

（2）Local policy implementation rate $\beta$: Extent to which local regulators adopt national standards (e.g., $\beta=0.75$ for Vietnam’s rural antibiotic supervision, reflecting constraints from limited rural regulatory resources).

3、Cross-case data validation
We will use hierarchical supervision data from three study scenarios—Saudi TB programs, Guangdong’s COVID-19 vaccination system, and Vietnam’s antibiotic oversight network—to test if the four-party model captures policy execution gaps (e.g., mismatches between national standards and local practice).

(6) PLOS authors have the option to publish the peer review history of their article ([what does this mean?](https://track.editorialmanager.com/CL0/https://journals.plos.org/plosone/s/editorial-and-peer-review-process%23loc-peer-review-history/1/010f0198bae474ba-1a416c12-825a-4e35-ae99-8b00d49f1c93-000000/ryR-Az1jREw9fD7oI13_EFmsKPOtEbPhQDgy4e4O6X0=224)). If published, this will include your full peer review and any attached files.
    If you choose “no”, your identity will remain anonymous but your review may still be made public.
    Do you want your identity to be public for this peer review? For information about this choice, including consent withdrawal, please see our [Privacy Policy](https://track.editorialmanager.com/CL0/https://www.plos.org/privacy-policy/1/010f0198bae474ba-1a416c12-825a-4e35-ae99-8b00d49f1c93-000000/ChGgBuC_SZJ52nxVEaYNh_OfS1AIm29oBN7GqhhrrVs=224).

Reviewer #1: No

I choose : No
